# Grail attenuates influenza A virus infection and pathogenesis by inhibiting viral nucleoprotein

**DOI:** 10.1038/s41598-018-35722-8

**Published:** 2018-11-22

**Authors:** Hui-Tsu Lin, Cheng-Cheung Chen, Pei-Yao Liu, Hsueh-Ling Wu, Ti-Hui Wu, Chih-Heng Huang, Ying-Chuan Chen

**Affiliations:** 1 Department of Physiology & Biophysics, National Defense Medical Center, Taipei, Taiwan 114 Republic of China; 2 Institute of Preventive Medicine, National Defense Medical Center, New Taipei City, Taiwan 114 Republic of China; 3 Division of Thoracic Surgery, Department of Surgery, Tri-Service General Hospital, National Defense Medical Center, Taipei, Taiwan 114 Republic of China

## Abstract

Grail is a well-characterized mediator of metabolic disease, tumour progression, and immune response. However, its role in influenza A virus (IAV) infection remains poorly understood. In this study, we demonstrated that *Grail* knockdown potentiates IAV infection, whereas Grail overexpression blocks IAV replication. The intranasal administration of IAV to *Grail* KO mice led to a lower survival rate than in similarly infected wild-type mice. Additionally, IAV-infected *Grail* KO mice had higher viral titres, greater immune cell infiltration, and increased expression of inflammatory cytokines in the lungs. Mechanistically, we showed that Grail interacts with viral nucleoprotein (NP), targeting it for degradation and inhibiting IAV replication. NP expression was increased in *Grail* knockdown cells and reduced in cells overexpressing *Grail*. Collectively, our results demonstrate that Grail acts as a negative regulator of IAV infection and replication by degrading viral NP. These data increase our understanding of the host antiviral response to infection with IAV.

## Introduction

Influenza A virus (IAV) is a highly feared pathogen that poses a significant threat to public health and holds the potential for worldwide outbreaks (pandemics). IAV belongs to the *Orthomyxoviridae* family of RNA viruses and contains a negative-sense, single-stranded RNA genome of 8 segments that encode 16 viral proteins on eight segments^1^. Replication of the IAV genome requires viral ribonucleoprotein (vRNP), which consists of the heterotrimeric, RNA-dependent RNA polymerase complex (PB1, PB2, and PA), oligomeric nucleoproteins (NPs), and viral RNA (vRNA). IAV vRNP plays a vital role in viral mRNA synthesis in the early stage of infection and genomic vRNA production later in infection^2^. Recent genome-wide RNAi screens and supporting experimental evidence have suggested that cellular host proteins interact with IAV at every stage of the viral life cycle^3–6^. Moreover, a number of host factors and cellular processes have been identified as potential regulators of vRNP function and thus may be implicated in host adaptation and viral pathogenicity^7–15^.

Increasing evidence has suggested that the host ubiquitin-proteasome system regulates key stages of the IAV life cycle. E3 ubiquitin ligases, of which there are over 600 types in human, predominantly control substrate specificity during ubiquitination^16^. Two of these, Itch and Nedd4, are involved in IAV uncoating from the endosome^6,17^, where the ubiquitinated viral capsids then activate and exploit the aggresome for efficient uncoating^18^. Several members of the tripartite motif (TRIM) superfamily have been reported to contribute to the host anti-influenza response by mediating proteasomal degradation of viral proteins; for example, TRIM22 and TRIM41 have been shown to ubiquitinate viral NP^19,20^, while TRIM32 conjugates polyubiquitin at the polymerase basic protein 1 protein^21^. However, the E3 ligase Ccr4-Not transcription complex subunit 4 (CNOT4) is another key host-derived mediator of NP ubiquitination that positively regulates viral RNA replication and does not lead to proteasomal degradation of NP^22^.

*Gene related to anergy in lymphocytes* (*Grail*) encodes a transmembrane protein that is involved in the expression of cytokines related to T cell activation. The deletion of *Grail* in mice leads to reduced T cell responsiveness under TCR stimulation^23,24^. Additionally, the mammalian target of rapamycin pathway has been shown to mediate the cell cycle progression and proliferation of naïve T cells through the regulation of *Grail* expression^25^. This evidence implies that Grail may play an important role in cell cycle arrest and proliferation. Our previous work also shows that Grail can regulate p53-mediated cell cycle arrest and apoptosis in response to DNA damage^26^. There is also evidence that Grail plays a crucial role in adipogenesis and diet induced obesity^27^. The dysregulation of Grail has been linked to ulcerative colitis, a chronic inflammatory disease of the gastrointestinal tract^28^. Recent work also indicates that Grail can regulate host innate immune responses to vesicular stomatitis virus (VSV) and herpes simplex virus type 1 (HSV-1) infections^29^. However, despite this body of research, the involvement of Grail during IAV infection and replication has not yet been well characterized. In the present study, we demonstrated that Grail aids in the control of IAV replication and infection by enhancing the degradation of viral NP *in vitro* and *in vivo*. Given this, we suggest that Grail is a potential drug target for the further prevention and treatment of IAV infections.

## Results

### *Grail* expression is upregulated after IAV infection *in vitro* and *in vivo*

**Figure 1:**
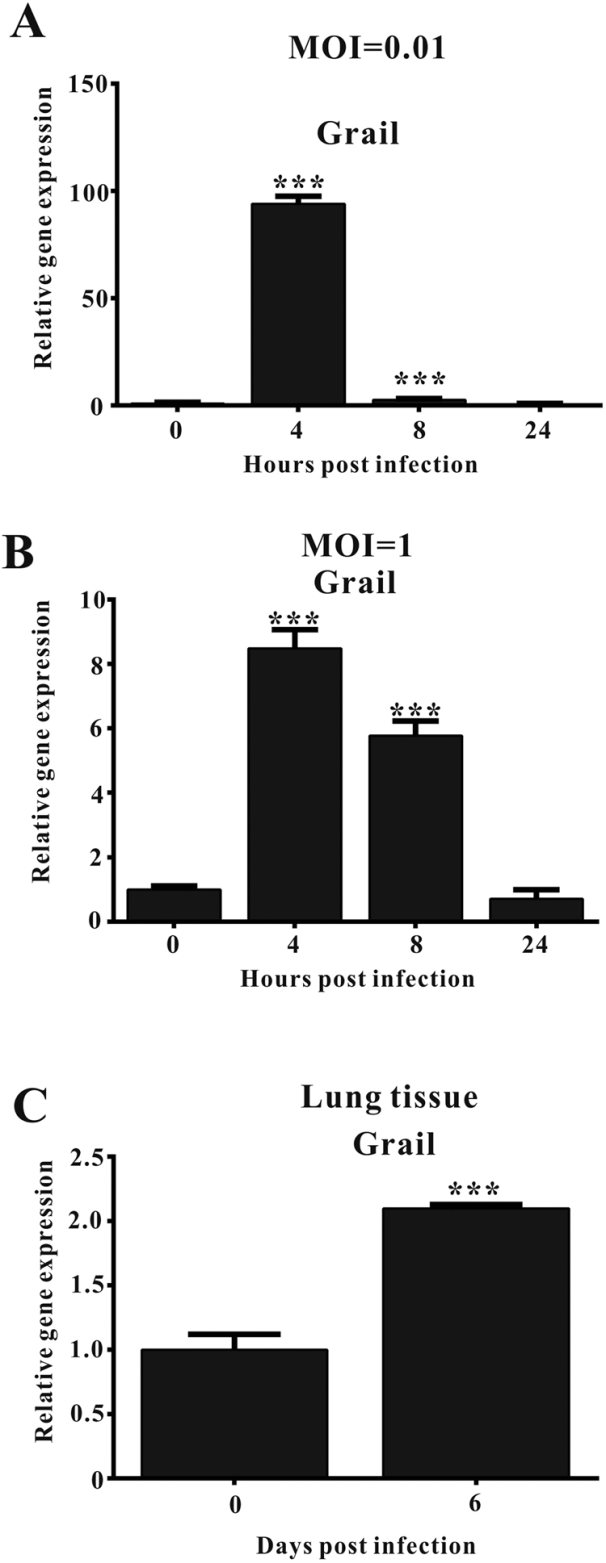
*Grail* expression is induced during IAV infection. (**A**,**B**) *Grail* expression in A549 cells infected with WSN virus at an MOI of 0.01 or 1 as determined by real-time PCR. (**C**) *Grail* expression in the lung tissue of mice infected with 1000 PFU of WSN. The data are presented as the mean ± SD and represent three independent experiments. ^***^P < 0.001.

Grail holds multiple biological roles in cell growth, adipogenesis, and immunity. However, the effect of Grail on the regulation of IAV infection is unclear. To address this, we first determined the expression level of *Grail* during IAV infection. *Grail* expression was shown to be induced in A549 cells infected with virus at an MOI of 1 (Fig. 1A and Supplementary Fig. 1) as well as in low titre infections using an MOI of 0.01 (Fig. 1B). Furthermore, mouse data showed that *Grail* expression was significantly higher in the lung tissue of animals infected with 1000 PFU of WSN than in control animals (Fig. 1C). These results show that IAV infection induces *Grail* expression.

### *Grail* knockout (KO) mice succumb to IAV infection

**Figure 2:**
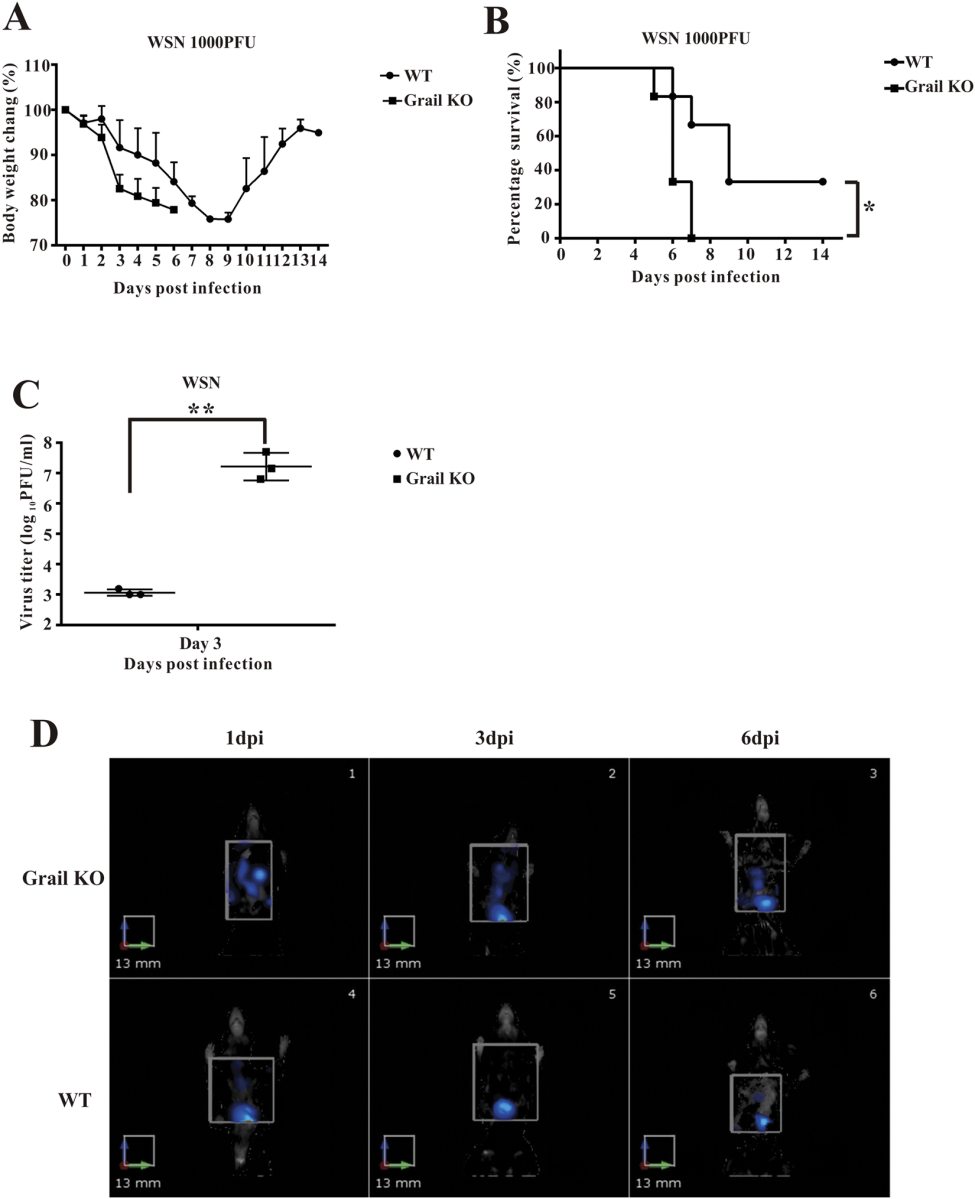
Grail restricts IAV pathogenesis *in vivo*. Grail knockout mice challenged intranasally with 1000 PFU of influenza A/WSN/33 (H1N1) (WSN) show more rapid disease progression and higher mortality than wild type mice. Body weight (**A**) and survival (**B**) of WSN-infected wild type and KO mice were monitored for 14 days after infection, revealing significant differences in survival (n = 6, p  = 0.0151). (**C**) Viral load in the lungs of animals challenged with 1000 PFU of WSN was measured at 3 dpi. Error bars show mean ± SD. (**D**) Near-infrared fluorescent *in vivo* imaging over time of WT and KO mice inoculated with 10^5^ PFU of H1N1 PR8-iRFP virus (n = 3). Brown colour indicates the most intense signal and blue the lowest.

Recently, it was shown that Grail improved innate antiviral immune responses to VSV (an RNA virus) and HSV-1 (a DNA virus) by enhancing TANK-binding kinase 1 (TBK1) activity^29^. To determine whether this antiviral property of Grail was also active against IAV infection *in vivo*, we employed mice carrying a deletion of the *Grail* gene. Wild-type (WT) and *Grail* KO mice were intranasally infected with 1000 PFU of mouse-adapted influenza A/WSN/33 H1N1 (WSN) virus. Both groups lost weight from day 2; however, *Grail* KO mice revealed a rapid period of weight loss at 3 dpi when compared to WT mice (Fig. 2A). In WT mice, WSN infection resulted in 66.6% mortality, while all *Grail* KO mice died by day 7 post-infection (Fig. 2B). The viral load in the lungs at 3 dpi, as determined by plaque assay, was 2 logs higher in the *Grail* KO mice than in the WT mice (Fig. 2C). Furthermore, we used the substrate-free IAV-iRFP reporter virus and strategy described by Fukuyama *et al*.^30^ to visualize the viral dynamics *in vivo*. We observed that iRFP fluorescence was detectable in mice infected with IAV-iRFP from 1 to 6 dpi. *Grail* KO mice demonstrated a wider distribution of this signal in the infected lung tissues than did WT mice (Fig. 2D). The iRFP signal was generally consistent with the viral load in the lungs of WSN-infected mice at 3 dpi (Fig. 2C,D). These *in vivo* data suggest that Grail may contribute to the regulation of influenza infection and replication, as these processes appear bolstered by the absence of functional Grail.

### Grail KO mice show more severe lung damage

**Figure 3:**
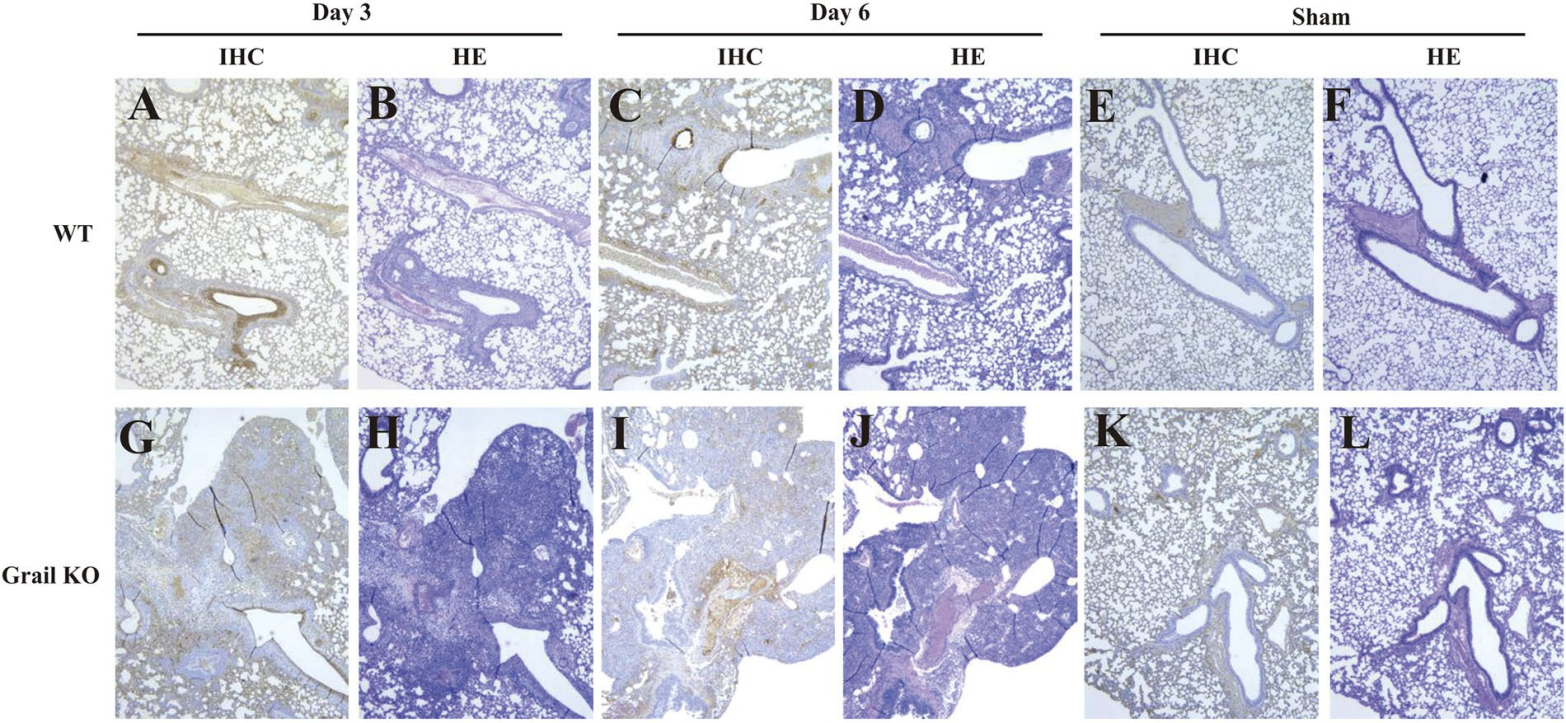
Histopathological findings in the lung of infected wild-type or KO mice. Representative histopathological images of WSN-infected lungs from WT or *Grail* KO mice at 3 and 6 dpi. Left panels show immunohistochemical staining for the detection of viral protein. Right panels show haematoxylin and eosin staining.

To further assess the causes of the increased susceptibility of *Grail* KO mice to WSN infection, histopathological examination of lung tissues collected at 3 and 6 dpi was performed. This revealed that both strains of mice developed viral pneumonia (Fig. 3B,D,H,J), in contrast to sham-infected mice (Fig. 3F,L). However, *Grail* KO mice had higher levels of multifocal acute alveolitis, with inflammatory cells infiltrating throughout the tissue, intra-alveolar oedema, red blood cell extravasation, epithelial necrosis (Fig. 3H,J), and higher histology scores than WT mice.

Immunohistochemistry further revealed differing levels of IAV antigen expression near the focal inflammation sites in lung sections (Fig. 3A,C,G,I). WSN-infected KO mice had higher levels and more widespread expression of viral antigen in the alveolar epithelium at 3 dpi (Fig. 3G). In contrast, WT mice displayed viral antigen expression most prominently in the bronchioles and had more limited expression in the alveolar epithelium (Fig. 3A).

### IAV infection induces increased inflammatory cytokine expression in Grail KO mice

**Figure 4:**
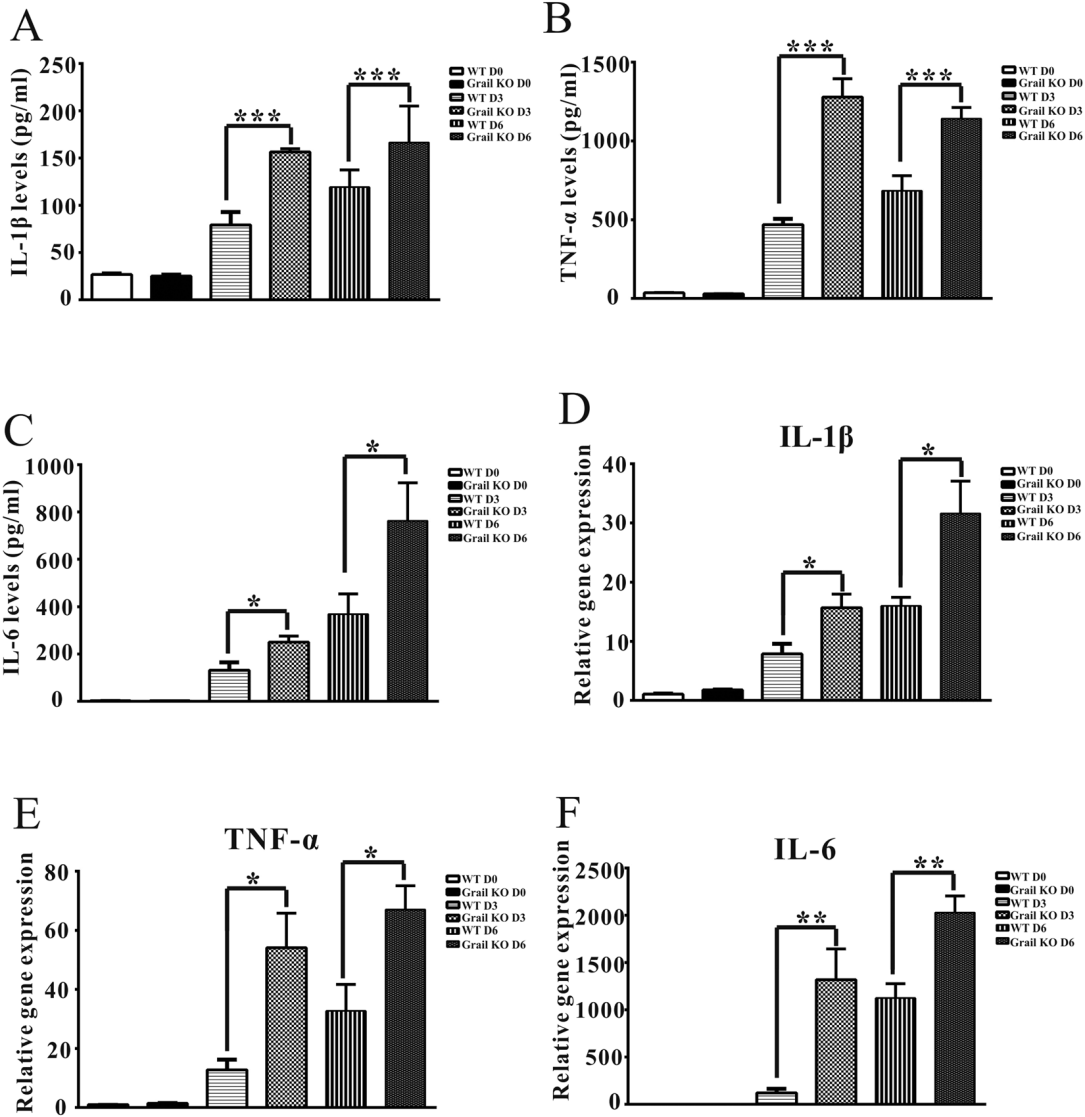
Inflammatory cytokine expression is reduced in IAV-infected *Grail* KO mice. (**A**–**G**) IL-1β, IL-6, and TNF-α mRNA and protein levels in the lung tissue of WT and *Grail* KO mice at 3 and 6 days post challenge with 1000 PFU of WSN. The data are presented as mean ± SD. ^*^P < 0.05; ^**^P < 0.01; ^***^P < 0.001.

To assess whether pulmonary responses to IAV infection in *Grail* KO mice differ from those of WT mice, we also determined the levels of inflammatory cytokines in the lung tissue of both types of infected mice. The mRNA and protein levels of TNF-α, IL-6, and IL-1β in the lung tissue of IAV-infected *Grail* KO mice were found to be significantly higher than those in similarly infected WT mice at 3 and 6 dpi (Fig. 4). Importantly, the expression of these cytokines did not differ between uninfected WT and *Grail* KO mice (Fig. 4).

### Grail suppresses viral replication

**Figure 5:**
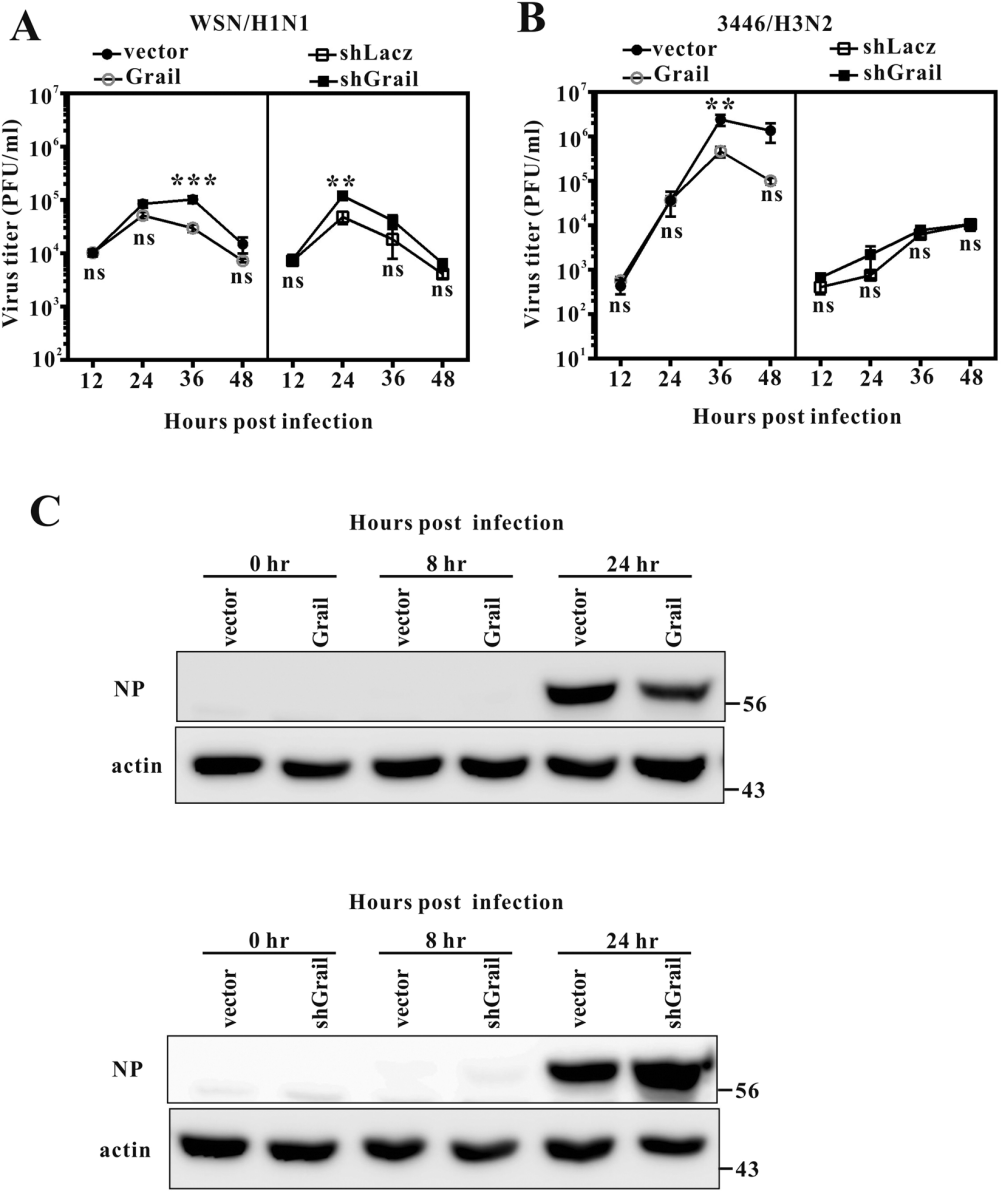
*Grail* overexpression inhibits IAV infection and *Grail* silencing increases IAV infection. Growth curve of WSN virus (**A**) or 3446 virus (**B**) in *Grail*-overexpressed or knockdown A549 cells. Cells were infected with WSN virus at an MOI of 0.01. Viral titres were determined by plaque assay in MDCK cells using cell supernatants collected at 12, 24, 36, and 48 hours post infection. NP protein levels (**C**) were analyzed in *Grail*-overexpressed or knockdown A549 cells at 0, 8, and 24 hours post infection. Data represent the mean ± SD from three independent experiments. ns, not statistically significant.

The data described thus far indicated that Grail may significantly enhance IAV-induced lung injury and lethality in mice. This prompted us to further investigate the functional relevance of Grail to IAV replication in human cells. We evaluated the growth kinetics of both WSN (an H1N1 influenza A virus) and 3446 (an H3N2 influenza A virus) in A549 cells under the conditions of either *Grail* overexpression or knockdown. We used shRNA to downregulate endogenous *Grail* and the retroviral transduction system for its stable overexpression, and examined the silencing or overexpressing efficacy (Supplementary Fig. 3). Upon downregulation of endogenous *Grail*, infected A549 cells had a significantly higher WSN titre at 24 hpi. Conversely, Grail overexpression led to a significantly reduced WSN titre at 36 hpi (Fig. 5A). While the growth curves of the 3446 virus showed no significant influence of Grail silencing in infected cells, the viral yield was significantly decreased at 36 hpi in A549 cells that overexpressed *Grail*, similar to the effect seen in WSN or pandemic/09 H1N1-infected cells (Fig. 5B and Supplementary Fig. 2). Furthermore, the expression of IAV NP protein correlated with the viral growth curves in the instances of both *Grail* knockdown or overexpression in cells infected with WSN at an MOI of 0.01 for 24 h, similar to the effect seen in 3446 virus-infected cells (Fig. 5C and Supplementary Fig. 4). These results clearly demonstrate that viral replication is suppressed by Grail.

### Grail regulates NP protein levels

**Figure 6:**
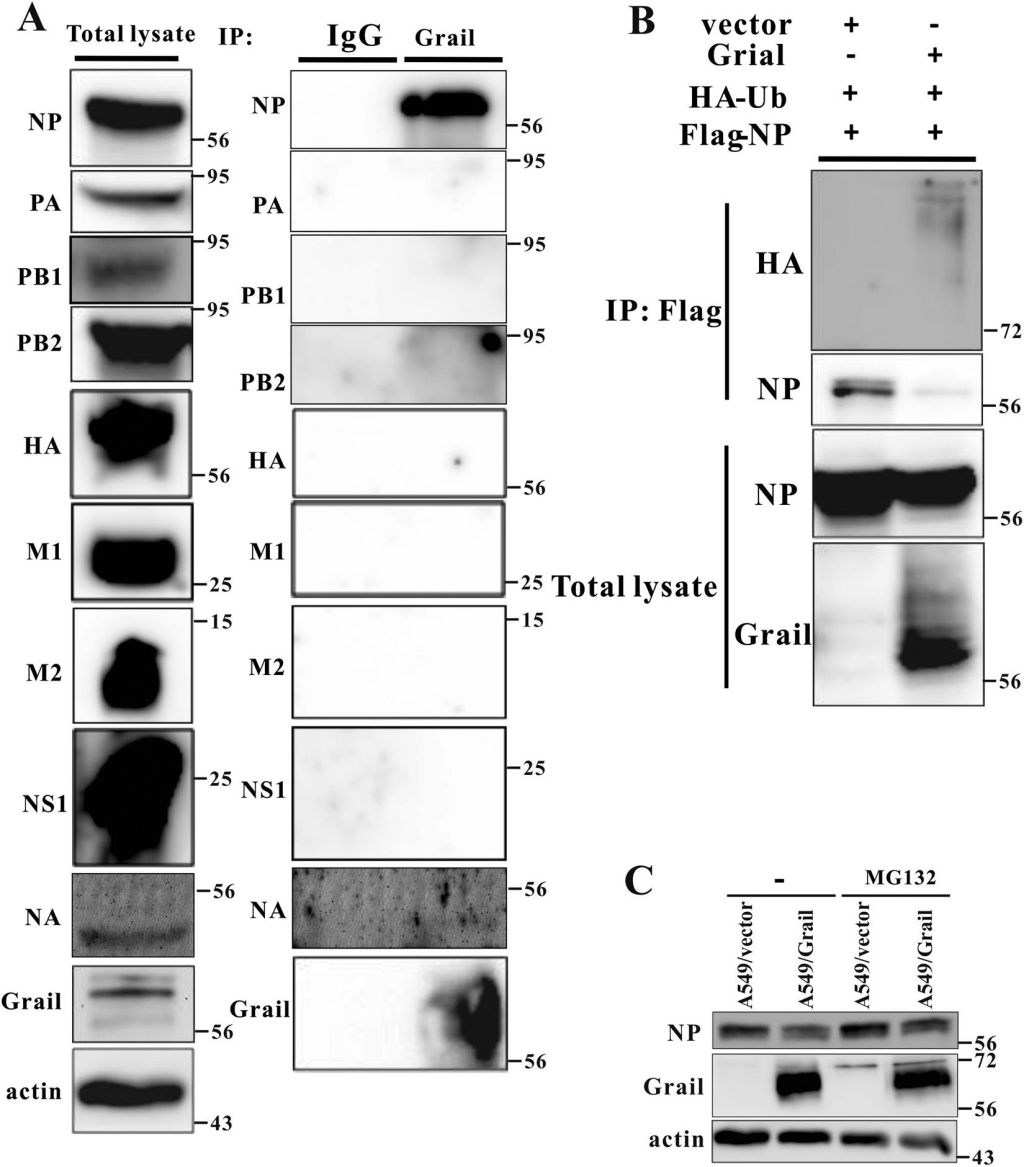
Grail interacts with NP and targets it for degradation. (**A**) Extracts from IAV-infected A549 cells were prepared, immunoprecipitated with anti-Grail or rabbit anti-IgG antibodies, and immunoblotted using the indicated antibodies, revealing interaction between Grail and viral NP. (**B**) Lysates from HEK-293 cells transiently transfected with HA-Ub, Flag-NP, and Grail expression plasmids were harvested and subjected to IP with anti-Flag antibody. Ubiquitination was observed by western blot, revealing a significant increase in NP ubiquitination when in the presence of Grail. (**C**) Cells were infected with IAV at an MOI of 0.1 in the presence or absence of MG132 for 24 hr. Immunoblot analysis shows that MG132 treatment rescues the NP depletion induced by overexpression of *Grail*.

Grail is an E3 ligase that functions by interacting with and degrading its targets. To elucidate the possible mechanism of Grail-mediated repression of IAV replication, we analysed whether Grail interacts with viral proteins (NP, PA, PB1, PB2, HA, NA, M1, M2 and NS1) and targets them for degradation. Lysates from IAV-infected A549 cells were treated with anti-Grail antibody and IP proceeded with NP, PA, PB1, PB2, HA, NA, M1, M2 or NS1 antibody. As shown in Fig. 6A, Grail interacted with NP but not PA, PB1, PB2, HA, NA, M1, M2 or NS1. In order to determine whether Grail mediates the ubiquitination of NP, we co-transfected Flag-NP and Grail into HEK293 cells and performed further IP experiments. These data showed that NP ubiquitination was significantly increased in the presence of Grail when compared with controls (Fig. 6B). To identify whether Grail targets NP for proteasome-mediated degradation, we transfected NP plasmids into A549/Vector and A549/Grail cells with or without MG132 treatment. In these cells, Grail overexpression reduced the NP protein level when compared to controls. However, NP expression was rescued in the presence of MG132 (Fig. 6C). These data provide evidence that Grail can interact with viral NP and subsequently target it for proteasome-mediated degradation.

## Discussion

The cellular ubiquitin-proteasome system impacts important steps of the IAV life cycle. Ubiquitination plays important roles in innate antiviral immunity by directly targeting viral proteins for degradation and activating antiviral signalling cascades^31^. On the other hand, recent studies have shown that IAV-associated ubiquitination promotes viral polymerase function independently of the proteasomal degradation of viral replication machinery (RNP complexes)^15,22^. Therefore, ubiquitination can be considered a double-edged sword in viral pathogenesis because it has both proviral and antiviral effects^32^. Influenza NP is abundant in both virions^33^ and infected cells^34^. NP plays key roles in the transcription and replication of the viral RNA genome as well as in the nuclear transport of vRNP during the life cycle of IAV^35^. NP is also one of main viral proteins to be targeted by host factors for posttranslational modifications such as phosphorylation, sumoylation, and ubiquitination^15^. Several recent studies on IAV-associated ubiquitin modifications have shown that NP ubiquitination can be catalysed by different E3 ligases, such as TRIM22, TRIM41, and CNOT4^19,22,36^. We found that the E3 ligase Grail is a host immune factor that restricts IAV infection directly through the ubiquitination of NP and its subsequent proteasomal degradation. Interestingly, it remains unclear how different E3 ligases can have the same substrate specificity for NP.

In this study, we demonstrated that Grail interacts with viral NP and targets it for degradation. As viral NP is required for the replication of viral RNA and thus also virus amplification, it follows that a loss of Grail would increase IAV replication by increasing the levels of NP, as has also been supported by experiments within this study. Conversely, the overexpression of Grail was shown to reduce the rate of virus replication by reducing the levels of NP. Our *in vitro* data were further supported by the *in vivo* observations that *Grail* KO mice had more immune cell infiltration, higher viral titres, and a greater level of inflammation in their lung tissue than WT mice following infection with IAV.

In a previous study, Grail expression was induced in peritoneal macrophages infected with Sev or HSV-1 virus and subsequently correlated with increases in TBK1 activation, thereby upregulating IRF3 activation and INF-β expression, ultimately leading to a more robust antiviral response^29^. Our data reveals an alternate antiviral mechanism of action by showing that Grail protects lung epithelial cells from IAV infection by enhancing the degradation of viral NP. Combining our data with the literature, we suggest that Grail exerts various antiviral effects through different mechanisms in a cell type-dependent manner. In future studies, it will be interesting to decipher the possible function of Grail in the cross-talk between macrophages and epithelial cells of the lung during IAV invasion.

In summary, we have found that Grail inhibits IAV replication through the promotion of NP degradation. However, it remains possible that, in addition to its influence of NP stability, Grail may interact with host proteins in ways that could also influence the viral infection process.

In conclusion, our findings provide evidence that Grail plays an important regulatory role during IAV infection and suggest that Grail may be a suitable target for the development of anti-influenza drugs.

## Materials and Methods

### Cell lines and viruses

HEK293, GP2-293, 293 T, A549, and MDCK cells were cultured in Dulbecco’s modified Eagle’s medium (DMEM) supplemented with 10% foetal bovine serum (FBS). The influenza viruses A/WSN/1933 (WSN, H1N1), pandemic/09 H1N1 and A/Taiwan/3446/2003 (3446, H3N2) were kindly provided by Professor Shin-Ru Shih (Chang Gung University, Taiwan). All viruses were propagated in MDCK cells in DMEM medium containing 1 µg/ml of L-1-tosyl phenylalanyl chloromethyl ketone (TPCK)-treated trypsin at 37 °C. IAV containing a near-infrared fluorescent protein (iRFP) reporter was generated by A/Puerto Rico/8/1934 (PR8, H1N1) using backbone 8-plasmid reverse genetics as described previously^37^. This strain (subsequently referred to as IAV-iRFP) was designed with the complete NS1 and NEP/NS2 genes flanking the iRFP reporter gene (iRFP702, Addgene). A Gly-Ser-Gly-Gly (GSGG) linker was inserted between the NS1 and iRFP genes, while a GSG linker and the 19 amino acid porcine teschovirus-1 (PTV-1) 2 A proteolytic sequence were inserted between the iRFP and NEP/NS2 coding sequences. In addition, two silent point mutations (525-CCCGGG-530) were introduced into the NS1 ORF in order to disrupt the endogenous splice acceptor site.

### cDNA constructs and transfection

Full length *Grail* and *NP* cDNA were cloned into the pCMVTNT (Promega) and pCMV-FLAG (Sigma-Aldrich) vector backbones, respectively. The 8-plasmid A/PR8/34 virus rescue system was kindly provided by Professor Shin-Ru Shih. The PR8 NS1-iRFP-2A-NEP reporter construct was synthesized by Genewiz and then cloned into pHW2000. *Grail* was cloned into the retroviral plasmid vector pQCXIP (Clontech). The pQCXIP-Grail and pQCXIP-empty plasmids were transfected into GP2-293 cells using TransIT-LT1. Transient gene expression was performed using TransIT-LT1 transfection reagent (Mirus Bio) according to the manufacturer’s instructions. Cells were plated and grown to 50–60% confluence prior to transfection. The cells were harvested and lysed in RIPA buffer (100 mM Tris-HCl pH 8.0, 150 mM NaCl, 0.1% SDS, and 1% Triton X-100) after transfection. To produce recombinant influenza A reporter viruses, the 3:1 co-cultured 293 T and MDCK cells were cotransfected with 1 μg of each plasmid encoding the 7 gene segments of the PR8 virus and the NS reporter construct using lipofectamine 2000 transfection reagent (Thermo) as previously described^37^.

### Immunoprecipitation and immunoblotting

Cells were harvested in lysis buffer (50 mM Tris pH 8.0, 5 mM NaCl, 0.5% NP-40, and 1X protease inhibitor), frozen and thawed three times, and then the proteins were recovered. Immunoprecipitation (IP) proceeded overnight at 4 °C in IP buffer containing antibodies against Grail or Flag. The IP mixture was then incubated with Dynabeads Protein G (Invitrogen) for 1 h prior to isolation using a DynaMag magnet and washing three times with SNNTE buffer (5% sucrose, 1% NP-40, 0.5 M NaCl, 50 mM Tris pH 7.4, and 5 mM EDTA). The immunoprecipitates were resuspended in SDS-PAGE sample buffer, boiled, and loaded onto a gel. Following separation, the proteins were transferred to a nitrocellulose membrane and the blot was probed with antibodies diluted in PBS/Tween 20 with 5% non-fat milk. Antibody detection was carried out using enhanced chemiluminescence reagents (GE Healthcare), as described by the manufacturer. The primary antibodies used for immunoblotting were: anti-PA (GeneTex), anti-PB1 (GeneTex), anti-PB2 (GeneTex), anti-HA (GeneTex), anti-NA (GeneTex), anti-NP (GeneTex), anti-M1 (GeneTex), anti-M2 (GeneTex), anti-NS1 (GeneTex), anti-HA (81B8, Cell Signaling, USA), anti-beta actin (MAb1501, Chemicon), and anti-Grail antibodies.

### Virus particle production, viral transduction, and RNA interference

Retroviruses were prepared according to the protocol published on the Clontech website. Oligonucleotides targeting the *Grail* sequence *5′-gaggcatccaagtcacaatgg-3′* were cloned into the retroviral shRNA expression vector pSIREN-Retro-Q (Clontech). Retroviruses expressing this *Grail* shRNA were generated according to the protocol published on the Clontech website. Cells were infected with retrovirus in selection medium supplemented with 2 μg/ml polybrene. After the infection, cells were treated with 2 μg/ml puromycin in order to positively select for the puromycin-resistant infected clones.

### Ethics statement

This study was carried out in strict accordance with the recommendations in the Guide for the Care and Use of Laboratory Animals of the National Institutes of Health (Taiwan). The protocol was approved by the Institutional Animal Care and Use Committee of National Defense Medical Center (Taipei, R.O.C., Taiwan) (ref no: IACUC-AN106-08) and all efforts were made to minimize suffering.

### Mouse experiments

All experimental animal procedures were approved by the Institutional Animal Care and Use Committee (ref no: IACUC-AN106-08) of the Preventive Medicine Institute, National Defense Medical Center, Taiwan. The Grail KO mice were generated by the Transgenic Mouse Models Core (Taipei, Taiwan) using CRISPR-Cas9 technology, which induced an NHEJ-mediated deletion in Grail exon 1, resulting in the removal of the first start codon, and the KO mice were generated on a C57BL/6 J background^27^. Six-week-old mice were used and randomly selected for the experimental and control groups. Mice were anesthetized intramuscularly with Zoletil 50 (25 mg/kg) and inoculated intranasally with 1000 PFU of virus in 50 μl of phosphate-buffered saline. The infected mice were weighed and observed daily for 14 days, noting any signs of illness or death. In accordance with institutional guidelines, mice that lost more than 25% of their initial body weight were considered moribund and thus scored as dead and euthanised. For the determination of lung titre, three mice from each group were euthanized at 3 days post-infection (dpi) and their lungs were collected for virological and pathological examination. Right lung samples were homogenized in 1.0 ml of unsupplemented DMEM using a Precellys 24 Tissue Homogenizer (Bertin). The homogenates were spun for 5 min at 2000 × g to remove cellular debris. Virus titres were determined by plaque assays in MDCK cells.

### Histopathology and immunohistochemistry

Three mice from each group were euthanised on days 4 and 8 post infection. Their left lungs were collected, fixed in 10% neutral-buffered formalin, and embedded in paraffin. Histopathological examination of sequential sections stained with haematoxylin and was performed. The MultiVision Polymer Detection System (Thermo) was used for antigen staining in serial sections. The primary antibody used for immunohistochemistry was a goat anti-influenza A antiserum (Chemicon AB1074, Millipore) that recognizes both the surface glycoproteins and internal proteins of the virus.

### *In vivo* imaging

Mice were anesthetized with 2% isoflurane and shaved before imaging. Mice infected with the iRFP reporter virus were imaged using the Fluorescence Molecular Tomography (FMT) 2000 Quantitative Tomography System (PerkinElmer, Waltham, MA, USA). Mice were placed in a biplanar FMT imaging cassette and carefully adjusted to ensure precise identification and repeat observation of the regions of interest (ROIs). Mice were imaged for 3–5 minutes using a 680 nm laser. Isoflurane anaesthesia was maintained for the duration of the imaging. Fluorescence intensity data were analysed and 3D images reconstructed through the use of the TrueQuant software package provided with the FMT2000.

### Real-time PCR

Total RNA from cells and tissues was isolated using TRIzol reagent (Sigma-Aldrich). Complementary DNA was synthesized using MMLV Reverse Transcriptase (Epicentre). Gene expression was determined using a Roche LightCycler 480. The primers used are listed in Supplementary Table 1.

### Cytokine assay

The expression of IL-1β, IL-6, and TNF-α in lung tissue lysates was determined using the Bio-Plex Multiplex Immunoassay kit (Bio-Rad) according to the manufacturer’s instructions. All assays were performed at room temperature in 96-well round-bottomed microtiter plates protected from light. Measurements and data analyses were performed with the Bio-Plex system in combination with Bio-Plex Manager software.

### Statistical analysis

Graphing and statistical analysis of data were performed using GraphPad Prism 7 (GraphPad Software). Kaplan-Meier survival curves were analysed using the Mantel-Cox log rank test for statistical significance. For comparison of multiple data sets, one-way analysis of variance (ANOVA) with Tukey’s multiple comparison was used. For analysis of two data sets, an unpaired two-tailed Student’s t-test was used. P values of ≤0.05 were considered statistically significant. (*P < 0.05; **P < 0.01; ***P < 0.001; ns, not significant).

## Electronic supplementary material

Supplementary Information
