# Supplementary material for: Grail attenuates influenza A virus infection and pathogenesis by inhibiting viral nucleoprotein
Source: Sci Rep. 2018 Nov 22;8:17242. doi: 10.1038/s41598-018-35722-8 (PMC6250720; doi:10.1038/s41598-018-35722-8)
Supplement: Supplementary file 1 — Supplementary Information [file 41598_2018_35722_MOESM1_ESM.docx]

**Supplementary Information**

**Grail attenuates influenza A virus infection and pathogenesis by inhibiting viral nucleoprotein**

**Hui-Tsu Lin^2,4^, Cheng-Cheung Chen^2,4^, Pei-Yao Liu^1,4^,**

**Hsueh-Ling Wu^2^, Ti-Hui Wu^3^, Chih-Heng Huang^2*^, Ying-Chuan Chen^1,2*^**

{Dominy, #450}

^1^Department of Physiology & Biophysics, National Defense Medical Center, Taipei, Taiwan 114, Republic of China; ^2^Institute of Preventive Medicine, National Defense Medical Center, New Taipei City, Taiwan 114, Republic of China ; ^3^Division of Thoracic Surgery, Department of Surgery, Tri-Service General Hospital, National Defense Medical Center, Taipei, Taiwan 114, Republic of China; ^4^These authors contributed equally

***Corresponding author:** Ying-Chuan Chen, Department of Physiology & Biophysics, National Defense Medical Center, 161, Sec. 6, MinChuan E. Rd. Room 6105, Taipei, Taiwan 114, Republic of China. Tel.: 886-2-87923100 ext 18599; Fax: 886-2-87923153; E-mail: addy0918@gmail.com; or Chih-Heng Huang, Institute of Preventive Medicine, National Defense Medical Center, New Taipei City, Taiwan 114, Republic of China. Tel.: 886-2-87923100 ext 19896; Fax: 886-2-26736954; E-mail: chin0096@gmail.com

**
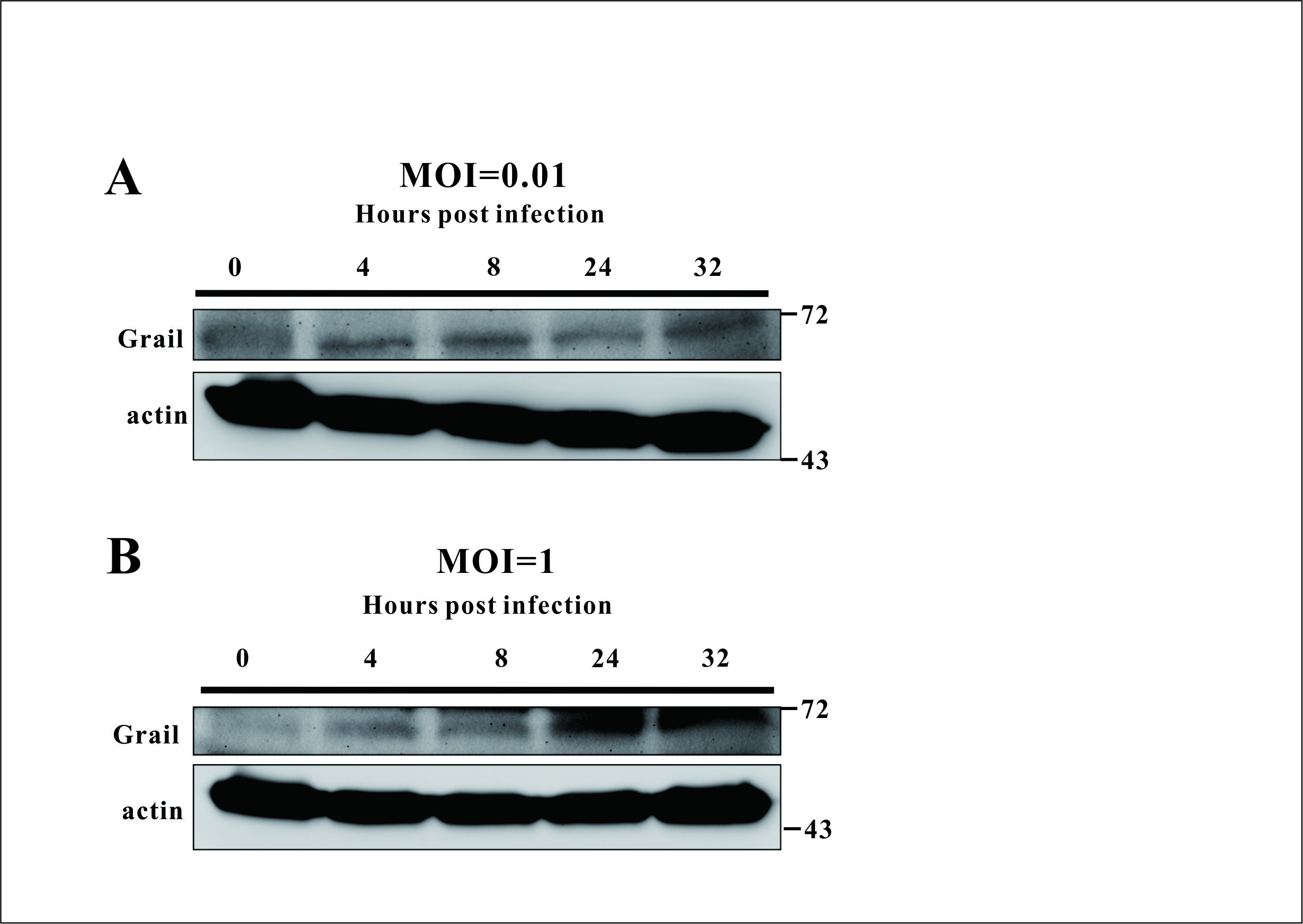
**

**Supplementary Fig. 1** Grail protein is induced during IAV infection.

(A-B) Grail protein expression in A549 cells infected with WSN virus at an MOI of 0.01 or 1 as determined by immunoblot.


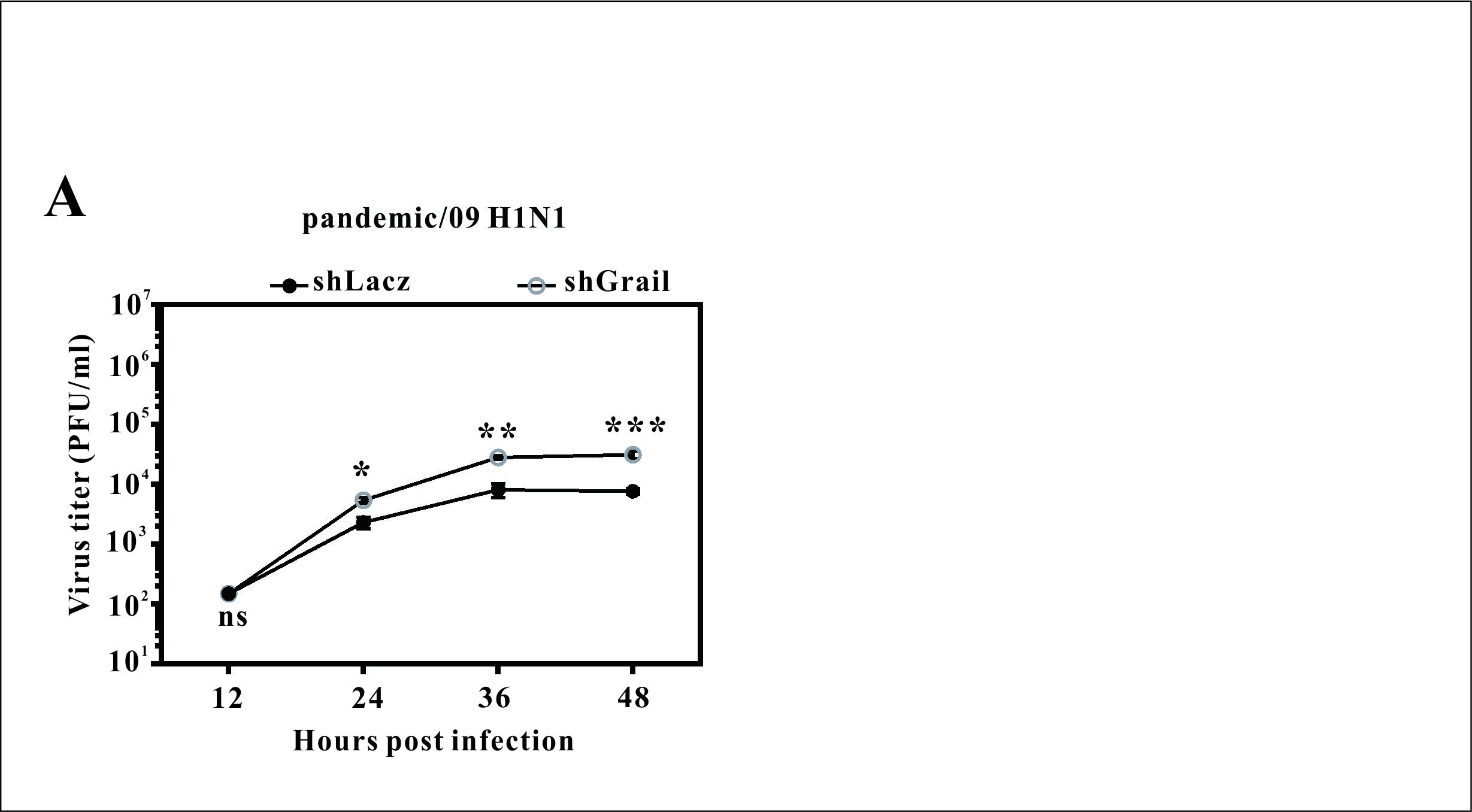


**Supplementary Fig. 2** *Grail* knockdown increases pandemic/09 H1N1 infection.

Growth curve of pandemic/09 H1N1 in *Grail*-knockdown A549 cells. Cells were infected with pandemic/09 H1N1 virus at an MOI of 0.1. Viral titres were determined by plaque assay in MDCK cells using cell supernatants collected at 12, 24, 36, and 48 hours post infection. Data represent the mean ± SD from three independent experiments. ns, not statistically significant. ^*^ *P* < 0.05; ^**^ *P* < 0.01; ^***^ *P* < 0.001


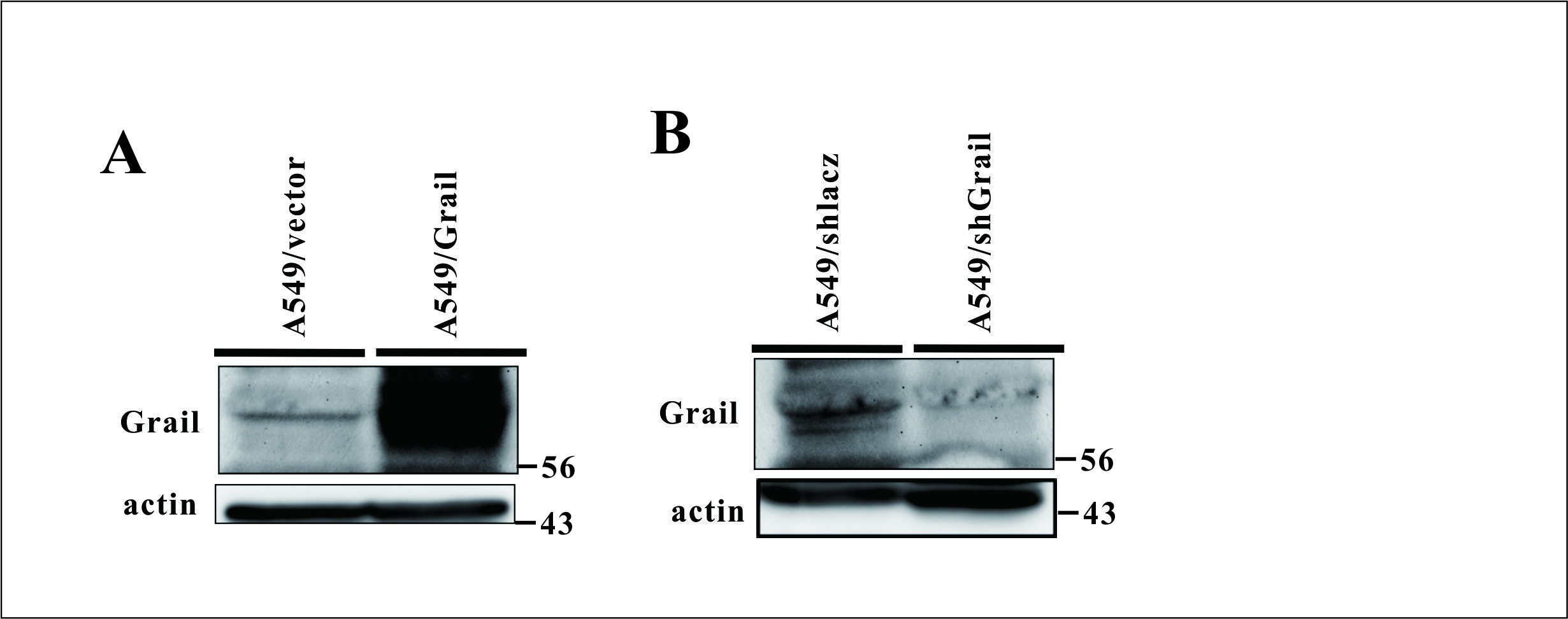


**Supplementary Fig. 3** Grail expression in knockdown or overexpression of Grail A549 cell lines.


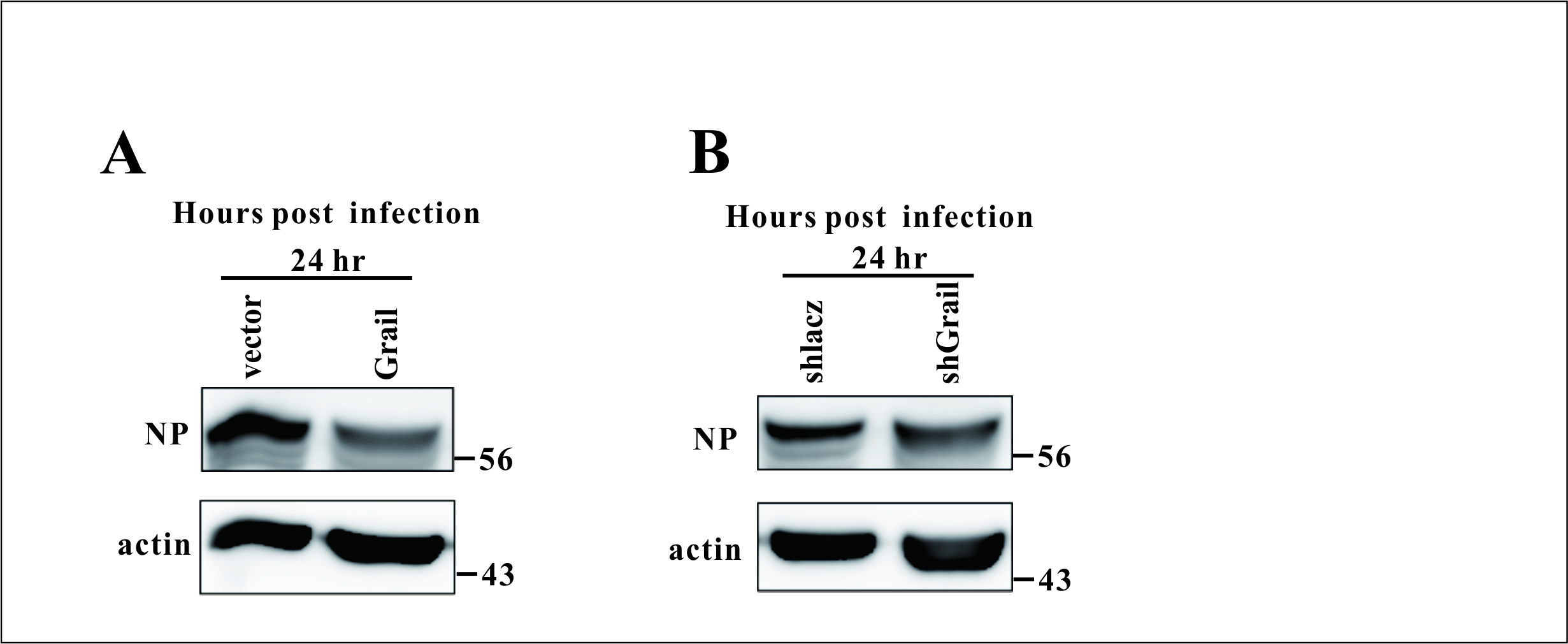


**Supplementary Fig. 4** Grail regulates NP protein levels in 3446 virus-infected cells.

Cells were infected with 3446 virus at an MOI of 0.01. The cell lysates were collected at 24 hours post infection and analyzed by immunoblot.

| **Supplementary Table 1 \| Primers used for Q-PCR analysis** | | |
| --- | --- | --- |
| **Primers** | **Forward sequence** | **Reverse sequence** |
| **Grail** | *5’-aaatgcaagagctcaaagcag-3’* | *5’-gcagctgaagctttccaatag-3’* |
| **IL-1β** | *5’-tgagcaccttcttttccttca-3’* | *5’-gcagctgtctaatgggaacg-3’* |
| **IL-6** | *5’-tctaattcatatcttcaaccaaga-3’* | *5’-tggtccttagccactccttc-3’* |
| **TNF-α** | *5’-cgagtgacaagcctgtagcc-3’* | *5’-ttgagatccatgccgttg-3’* |
| **actin** | *5’-ctaaggccaaccgtgaaaag-3’* | *5’-accagaggcatacagggaca-3’* |
